# Antenatal Hypoxia and Programming of Glucocorticoid Receptor Expression in the Adult Rat Heart

**DOI:** 10.3389/fphys.2019.00323

**Published:** 2019-04-02

**Authors:** Juanxiu Lv, Qingyi Ma, Chiranjib Dasgupta, Zhice Xu, Lubo Zhang

**Affiliations:** ^1^Institute for Fetology, The First Affiliated Hospital of Soochow University, Suzhou, China; ^2^Lawrence D. Longo, MD Center for Perinatal Biology, Department of Basic Sciences, Loma Linda University School of Medicine, Loma Linda, CA, United States

**Keywords:** hypoxia, glucocorticoid receptor, programming, heart, DNA methylation, sex

## Abstract

Glucocorticoid receptor (GR) signaling is critical for development and function of the heart. Our previous study demonstrated that gestational hypoxia induced epigenetic repression of the GR gene in the developing heart. The present study aims to determine that the alterations of promoter methylation level and epigenetic repression of the GR gene in the developing heart in response to maternal hypoxia is sustained in adult offspring and potential gender differences in the programming of GR gene. Pregnant rats were treated with 10.5% O_2_ from gestational day 15 (E15) to 21 (E21). Hearts were isolated from 5-month-old male and female offspring with the developing stage being equivalent to 18-year-old human. GR mRNA and protein abundance was determined with real time qRT-PCR and Western blot. GR gene promoter methylation and binding of transcription factors were measured with methylated DNA immunoprecipitation (MeDIP) and Chromatin immunoprecipitation (ChIP). The results showed that antenatal hypoxia significantly decreased the expression of GR mRNA and protein in the hearts of adult male offspring, but not in females, which is ascribed to the differential changes of alternative exon1 mRNA variants of GR gene in male and female hearts in response to prenatal hypoxia. In addition, the downregulation of GR expression in the male heart was correlated with increased methylation levels of CpG dinucleotides in promoters of exon 1_4_, 1_5_, 1_6_, 1_7_, and 1_10_, which resulted in a decrease in the binding of their transcription factors. Thus, the study reveals that antenatal hypoxia results in a reprogramming and long-term change in GR gene expression in the heart by hypermethylation of GR promoter in a sex-differential pattern, which provides a novel mechanism regarding the increased vulnerability of heart later in life with exposure of prenatal hypoxia.

## Introduction

Numerous human and animal studies have shown that prenatal and early neonatal environments change developmental trajectories of the heart and contribute to the individual life-long health problems such as cardiology disorders (Barker, 2004; Gluckman and Hanson, 2004; Dasinger and Alexander, 2016; Morton et al., 2016). Hypoxia is one of the most common consequences in complicated pregnancy (Hutter et al., 2010; Giussani and Davidge, 2013). The clinical signs include insufficient uterine blood flow, increased placental vascular resistance, reduced umbilical cord blood flow and decreased oxygen level. In clinical practice, anemia or increased fetal oxygen consumption may lead to fetal hypoxia. The fetus is able to make a series of adaption to hypoxia challenge, which helps to ensure its survival. However, this “developmental plasticity” may cause health problems in adulthood (Nathanielsz, 2006). Indeed, the theory of developmental origin of health and disease (DOHaD) proposes that vulnerabilities of human to chronic maladies, to some extent, depend on physiological changes in the womb in response to environmental changes during the prenatal period (McMillen and Robinson, 2005). There is a common notion that intrauterine hypoxia can induce long term “stress response” (Giussani et al., 2014; Ramamoorthy and Cidlowski, 2016). Recent progress has been made in demonstrating the cardiovascular disorder in adult offspring following prenatal hypoxia. Rook et al. (2014) reported that fetal programming induced by prenatal hypoxia led to increased sympathetic activity in the central nervous system and hypertension in middle-age rats. A study from Camm et al. (2011) using rodents suggested that prenatal hypoxia was linked to the insulin resistance in adult offspring. We also determined that prenatal hypoxia increased the susceptibility of adult offspring to cardiac ischemia/reperfusion injury in rats, which was associated with a decreased expression of PKC𝜀, a cardiac protective gene. Mechanistically, further study revealed that promoter hypermethylation of PKC gene was the primary mechanism responsible for the downregulation PKC gene in rat heart after prenatal hypoxia, which was reversed by DNA methylation inhibitors (Patterson and Zhang, 2010).

In addition to PKC gene, glucocorticoid profoundly affects the development and maturity of the cardiovascular system (Waffarn and Davis, 2012; Xiong and Zhang, 2013; Xiong et al., 2016). This hormone exerts its functions by activation of glucocorticoid receptor (GR, *NR3C1*) (Oakley and Cidlowski, 2015). Altered GR signal system is associated with cardiovascular diseases (Seremak-Mrozikiewicz et al., 2014). It has been determined that GR pathway is programmed in response to prenatal adverse environments, and such programming changes have been observed in adulthood (Speirs et al., 2004; Hadoke et al., 2009; Giussani and Davidge, 2013; Bellisario et al., 2015; Caldwell et al., 2015; Anwar et al., 2016). All of this evidence suggests that GR plays a key role in heart development and functions, and the GR reprogramming in the early stage of life may affect the health in adulthood (McCormick et al., 2000; Reynolds, 2013; Rog-Zielinska et al., 2013). It has been documented that the changes in DNA methylation levels of GR gene play an important role in regulating the effect of glucocorticoid in fetal growth, tissue development and blood pressure. Our previous study demonstrated that prenatal hypoxia suppressed the expression of GR gene via promoter hypermethylation in the developing heart, ultimately leading to the development of ischemia-sensitive phenotype in offspring (Xiong et al., 2016). However, whether reprogramming of GR in the developing heart could continue into adulthood remains unknown.

In the present study, we investigated the epigenetic regulation of GR gene in the heart of adult offspring in response to prenatal hypoxia. We found that prenatal hypoxia significantly decreased the expression of GR in the hearts of adult male offspring, but not females. Furthermore, the downregulation of GR gene in the male heart was correlated with increased methylation levels of CpG dinucleotides in alternative promoters of exon 1 and decreased the binding of transcription factors CREB and SP1 to promoters of exon 1_4_, 1_5_, 1_6_, 1_7_, and 1_10_. The present study sought to explore the mechanistic link between prenatal hypoxia and the GR reprogramming in adult offspring in rats.

## Materials and Methods

### Experimental Animals

Time-dated pregnant Sprague-Dawley rats were purchased from Charles River Laboratories (Portage, MI, United States). Pregnant rats were randomly divided into normoxic (21% O_2_) and hypoxic (10.5% O_2_) group from E15 to E21 as previously described (Xiong et al., 2016). When offspring grow to 5 months old, rats were anesthetized with isoflurane (5% for induction, 2% for maintenance) in oxygen (2 L/min for induction, 1 L/min for maintenance) and hearts were removed. The left ventricle of the heart was isolated, immediately frozen in liquid nitrogen, and stored at -80°C until analysis. About 10 dams were used for this study. Each experiment group included offspring from at least two litters. To avoid the potential effect of litter size on the result, only the litters with 8–10 pups were used. All procedures and protocols were approved by Institutional Animal Care and Use Committee of Loma Linda University. All experiments followed the guidelines of the National Institutes of Health Guide for the Care and Use of Laboratory Animals.

### Western Blot

Heart tissue was extracted for protein using RIPA lysis buffer (Life Technologies, Carlsbad, CA, United States) and proteinase inhibitors cocktail (Pierce Biotechnology, Rockford, IL, United States). Protein concentrations were measured using the BCA kit (Pierce). Samples with equal amounts of proteins were separated by SDS-PAGE and transferred onto Immobilon-P membranes (Millipore Corporation, Billerica, MA, United States). After blocking with 5% non-fat milk in TBS (blocking buffer) for 2 h at room temperature, membranes were then incubated with primary antibodies against GR (1:1000; sc-1002; Santa Cruz Biotechnology, Santa Cruz, CA, United States) or β-actin (1:6000; A5316; Sigma-Aldrich, St. Louis, MO, United States) in blocking buffer at 4°C overnight followed by secondary antibody (1:4000; Santa Cruz Biotechnology) for 1 h at room temperature. The bands were visualized using Amersham ECL^TM^ Western Blotting Detection Reagents (GE Healthcare, United States), and the blots were exposed to Hyperfilm. The results were analyzed using the Kodak ID image analysis software. GR protein abundance was normalized to β-actin.

### Real-Time RT-PCR

TRIzol reagent (Life Technologies) was used for isolating heart tissue RNA. SuperScript First-Strand Synthesis Kit was used for reverse transcription reaction (Life Technologies). Primers used were listed in Table 1. A real-time PCR reaction mixture was 25 μl. The following PCR program on iQ SYBR Green Supermix (Bio-Rad, Hercules, CA, United States) was used: 95°C 10 min, 1 cycle, 95°C 15 s, 60°C 30 s, 72°C 30 s, 40 cycles, and 72°C 10 min, 1 cycle. PCR was conducted in triplicates and averaged threshold cycle numbers were accepted for each sample. The specificity was examined using 3% agarose gel with 5 μl products from each reaction.

**Table 1:** Primer sequences.

	Forward	Reverse
exon1.4	AAGCAACACCGTAACACCTT	AGAAGCAGCAGCCACTGA
exon1.5	CATGCAACTTCCTCCGAGT	
exon1.7	GGAGCCTGGGAGAAGAGAAA	
exon1.11	GCCGCAGAGAACTCAACAG	
exon1.10	CACGCCGACTTGTTTATC	TCTGCTGCTTGGAATCTG
exon1.6	ACCTGG CGG CAC GCG AGT	GCAGCCACTGAGGGCGAAGA
exon1.9	GTCAGTGCCTGGAGCCCGAG	AGCAGCCACTGAGGGCGAAG
GR	AGGTCTGAAGAGCCAAGAGTTA	TGGAAGCAGTAGGTAAGGAGAT
actin	TCAGGTCATCACTATCGGCAAT	ACTGTGTTGGCATAGAGGTCTT
GR-IP-1.4	AAAGAACGACTCGGGTTTGA	CTCTGCCTGACCTCTTGGAG
GR-IP-1.5	ACAGCTGGACGGAGCTAAAA	CCCGAATCTTGACATTTGCT
GR-IP-1.6	GGGTTCTGCTTTGCAACTTC	GAGAGGGTCAGCGCATACAT
GR-IP-1.7	GACACACTTCGCGCAACTC	CACCCAAGGAACGAGAAAAA
GR-IP-1.10	GGGACGGATTCTAAGTGGGT	AGATAAACAAGTCGGCGTGC


### Methylated DNA Immunoprecipitation (MeDIP)

Methylated DNA Immunoprecipitation assay was performed with the MeDIP kit (Active Motif) following the manufacturer’s instructions. Briefly, genomic DNA was extracted from heart tissue and purified by standard procedures. Genomic DNA was sheared through sonication to produce random fragments between 200 to 1,000 bp. DNA fragments were denatured at 95°C in order to yield single stranded DNA fragments and incubated with the 5-mC antibody to precipitate DNA containing 5-mC, which was captured by the magnetic beads. The 5-mC antibody pull-down DNA and input DNA were then purified with phenol/chloroform extraction and subjected to quantitative real-time PCR analysis. Sequence of primers flanking the appropriate GR promoters was listed in Table 1.

### Chromatin Immunoprecipitation (ChIP)

Chromatin Immunoprecipitation assay was performed using the Chip-IT Express Kit (Active Motif) as previously described (13, 26). Briefly, tissues were minced and protein-DNA complexes were fixed with 1.5% formaldehyde. Tissues were then homogenized in lysis buffer (10 μg/mL Leupeptin, 10 μg/mL Aprotinin, and 1 mM PMSF) on ice. Samples were sonicated to shear chromatin to an average length between 200 and 1,000 bp. After incubation with antibodies against CREB (binding site at – 4408 and – 3896) (#4820; Cell Signaling, Danvers, MA, United States) or Sp1 (binding site at – 3425 and – 3034) (39058; Active Motif), magnetic beads (50 μl) were added into the samples. Crosslinking was then reversed using a salt solution and proteins were digested with proteinase K. The antibody-pulled chromatin extracts were then subjected to real-time quantitative PCR analysis using two primers that flank the predicted transcription factor binding sites at GR promoters, as described above in MeDIP (Xiong et al., 2016).

### Statistical Analysis

Statistical analysis was performed with repeated-measures ANOVA (MANOVA) followed by Tukey *post hoc* test or Student’s *t*-test (unpaired, 2-tailed), where appropriate. All data were expressed as means ± SEM, and statistical significance was set at *p* < 0.05. Statistical analyses were performed using GraphPad Prism software.

## Results

### Prenatal Hypoxia Sex-Differentially Regulated GR Expression in the Heart of Adult Offspring

**FIGURE 1:**
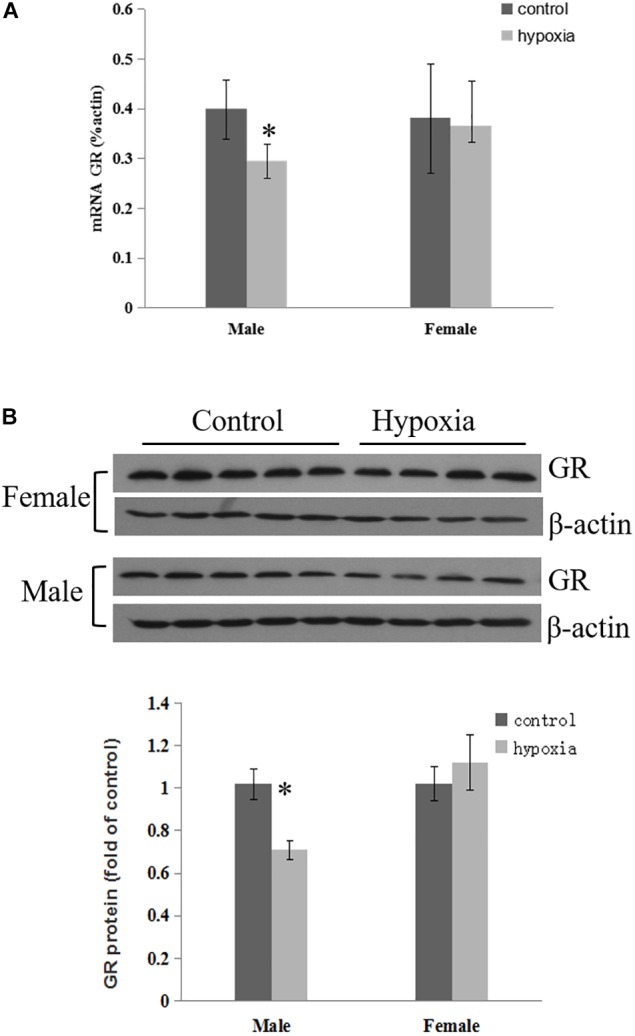
Maternal hypoxia decreased the expression of GR protein and mRNA. Left ventricle of hearts was isolated from 5-month-old male and female offspring exposed to normoxia or hypoxia from E15 to E21. **(A)** GR mRNA abundance was determined by quantitative real-time RT-PCR. **(B)** GR protein abundance was determined using Western Blot analysis. Data are mean ± SEM, *n* = 4–5 (^∗^*p* < 0.05, hypoxia vs. control).

The effect of prenatal hypoxia on GR expression in the heart was detected in male and female adult offspring. The abundance of cardiac GR mRNA and protein was significantly decreased in male adult offspring, but not females, with exposure of prenatal hypoxia, as compared to normoxic controls (Figure 1A,B). This data suggests that prenatal hypoxia causes a sex-differential regulation of GR expression in the hearts of adult offspring.

### Prenatal Hypoxia Sex-Differentially Altered GR Alternative Exon 1 mRNA Variants in the Hearts of Adult Offspring

**FIGURE 2:**
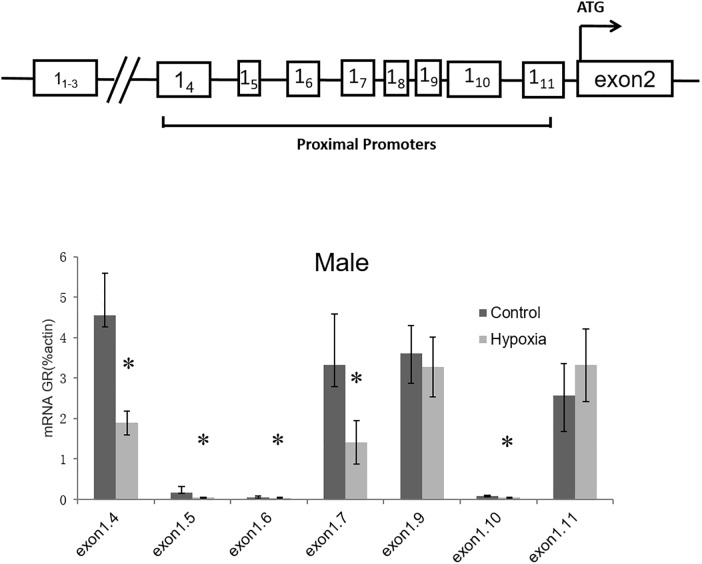
Effects of prenatal hypoxia on the expression of GR exon 1 variants in the hearts of adult male and female offspring. Up panel, diagrammatic representation of GR gene structure. The mRNA abundance of GR exon 1 variants of the 5-month-old male was determined by quantitative real-time RT-PCR. Data are mean ± SEM, *n* = 5 (^∗^*p* < 0.05, hypoxia vs. control).

Considering that GR total mRNA consists of transcripts containing multiple exon1 variants, we further determined the effect of prenatal hypoxia on the transcriptions of GR variants. Since GR exon1_1-3_ was not expressed in the heart (McCormick et al., 2000), our following experiments focused on the proximal exon 1_4-11_. Quantitative RT-PCR was carried out using primers designed to amplify specific transcripts containing GR exon 1_4_ to exon 1_11_. For each set of primers, forward primers were located in the exon1, while reverse primers were located in the common exon 2 region. The result showed that transcripts of exon 1_4_, 1_5_, 1_6_, 1_7_, and 1_10_ were significantly decreased in the heart of male adult offspring with exposure of prenatal hypoxia. Among all exon 1 mRNA variants, the 1_4,_ 1_7_, 1_9_, and 1_11_ containing mRNA is predominantly expressed in the heart of adult males (Figure 2).

### Prenatal Hypoxia Increased the Promoter Methylation Levels of GR Alternative Exon 1 mRNA Variants in the Hearts of Male Offspring

**FIGURE 3:**
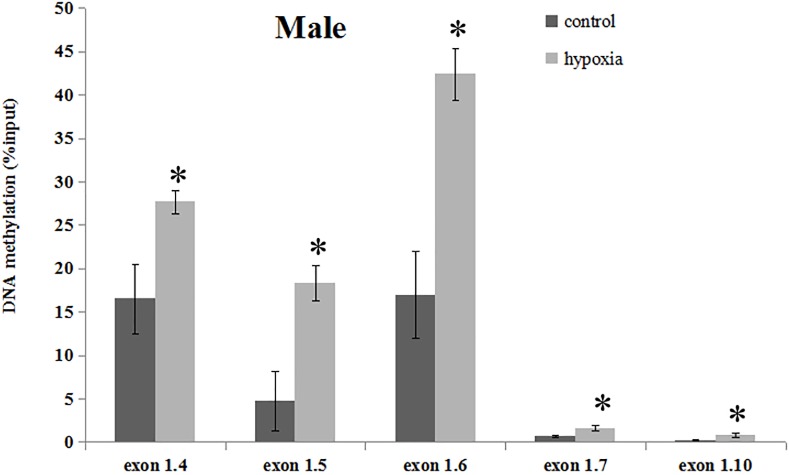
DNA methylation levels of GR exon 1 promoters in the hearts of male adult offspring. Methylation of promoter region of GR exon 1_4_, 1_5_, 1_6_, 1_7_, and 1_10_ in the heart was determined by MeDIP. F, forward primer, R, reverse primer. Data are mean ± SEM, *n* = 5 (^∗^*p* < 0.05, hypoxia vs. control).

Our previous study has determined that prenatal stress affects GR gene expression through hypermethylation of GR promoter (Xiong et al., 2016). Therefore, we next determined the effect of prenatal hypoxia on the methylation levels of the GR promoter 1_4_, 1_5_, 1_6_, 1_7_, and 1_10_ by precipitation of methylated DNA and subsequent PCR analysis in the heart of adult males. As shown in Figure 3, the general methylation levels of promoter 1_4_, 1_5_, 1_6_, 1_7_, and 1_10_ were significantly increased in adult males exposed to prenatal hypoxia, as compared to normoxic control.

### Prenatal Hypoxia Decreased the Binding of Transcription Factors to GR Alternative Exon 1 Variant Promoters

**FIGURE 4:**
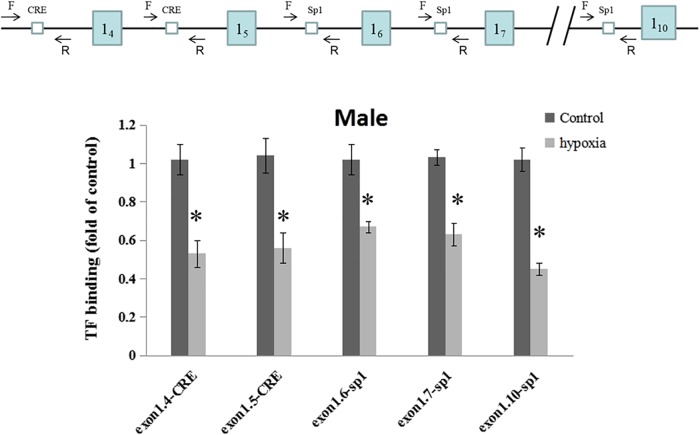
Effects of prenatal hypoxia on the binding of CREB and Sp1 to GR exon 1 promoters in the hearts of male adult offspring. Up panel, diagrammatic representation of the putative transcription factor binding sites at GR promoter and the relative location of primer set. Binding of CREB and Sp1 to GR promoter was determined by ChIP assay. Data are mean ± SEM, *n* = 5 (^∗^*p* < 0.05, hypoxia vs. control).

The regulating region of GR gene is embedded in a CpG island with many CpGs that could potentially be methylated. Multiple putative transcription factor binding sites with CpGs were found at the GR promoter, including CREB (cAMP response element-binding proteins) response elements and Sp1 binding sites. To investigate the *in vivo* effect of hypoxia-caused hyper-methylation in GR regulation, the binding of CREB to GR promoter 1_4_ and 1_5_, and the binding of Sp1 to promoter 1_6_, 1_7_, and 1_10_ were further determined in the male offspring heart by ChIP assay. As shown in Figure 4, prenatal hypoxia significantly decreased the binding of CREB to GR promoter 1_4_ and 1_5_, as well as reduced the binding of Sp1 to promoter 1_6_, 1_7_, and 1_10_.

## Discussion

Our previous study has determined that prenatal hypoxia results in downregulation of GR expression mainly *via* promoter hypermethylation in the developing heart. In the present study, we found that the expression of GR was significantly reduced in the heart of adult offspring exposed to prenatal hypoxia and the downregulation of GR gene showed a sex-differential pattern. Moreover, we determined that epigenetic modification was the major mechanism in regulating GR expression in response to prenatal hypoxia by showing increased methylation of CpG dinucleotides in the GR promoters of exon 1_4_, 1_5_, 1_6_, 1_7_, and 1_10_, which caused the decrease of transcription factor binding activities. This study advances our understanding of the role of GR reprogramming in the development of ischemic-sensitive phenotype in the heart of adulthood.

Maternal stress has been increasingly recognized for its important contribution to the development and progression of cardiac diseases in later life. The hypothalamic-pituitary-adrenal axis (HPA) is activated in response to stress, and then results in the release of glucocorticoids from the adrenal gland (Waffarn and Davis, 2012; Xiong and Zhang, 2013; Xiong et al., 2016). As the primary stress hormone in humans, glucocorticoids act on numerous target tissues to regulate a plethora of biological processes. The physiological and pharmacological actions of glucocorticoids are mediated by GR, a member of the nuclear receptor super family of ligand-dependent transcription factors (Leonard et al., 2005; Turecki and Meaney, 2016; Vitellius et al., 2016). A recent study using transgenic mice revealed that GR signaling was critical for the normal development and functions of the heart (Oakley et al., 2013). Moreover, GR in peripheral tissue plays a critical role in the development of chronic diseases (Inoue et al., 2016; John et al., 2016; Kaya et al., 2016; Lee et al., 2016). We have determined that GR downregulation promotes the development of ischemic-sensitive phenotype in the developing heart, leading to increased vulnerability of the heart in offspring to ischemia/reperfusion injury (Inoue et al., 2016). Following this study, the present study further demonstrated that GR protein and mRNA levels were decreased in the heart of adult offspring following prenatal hypoxia. Moreover, repression of the GR gene in adult offspring was through a gender-difference pattern showing a significant downregulation of GR expression in male adult offspring, but not females. This finding is in consistence with the notion that females and males showed different response in the pathophysiology of cardiovascular diseases (Regitz-Zagrosek, 2006; Ostadal et al., 2009), and the heart of females had higher tolerance to cardiac injury following ischemia/reperfusion insult than males (Murphy and Steenbergen, 2007). It has been documented that female sex hormone estrogen may play a key role in the sex-differential response to cardiac injury, which has been reported to reduce the risk of ischemic heart disease and provide cardioprotective effect in myocardial ischemia (Giardina, 2000; Ross and Howlett, 2012). However, as a key hormone in response to stress, glucocorticoids also protected against myocardial ischemia/reperfusion injury (Filep, 2014). Thus, our finding provided a new mechanism in explaining the sex-differential resistance to heart insult.

We next focused on the expression of GR gene by targeting exon 1 promoter, which has multiple alternatives responsible for GR expression in response to fetal hypoxia. McCormick et al. (2000) demonstrated that GR exon 1_s_ were differentially expressed at variant levels in tissues of adult rats. Among the relatively abundant GR exon 1_s_, exon 1_10_ was found to be accounting for at least half of total GR transcripts while exon 1_6_ contributed about 10–20% of total GR mRNA in the adult heart. Our previous study has detected the expression of all proximal GR exon 1_s_, except for exon 1_8_, in the near term fetal rat heart after prenatal hypoxia, and found a significant decrease of GR exon 1_4_, 1_5_, 1_6_, and 1_7_ transcripts, leading to reduced GR levels in the fetal heart (Xiong et al., 2016). In the present study, using real-time PCR that is both sensitive and quantitative, we found that prenatal hypoxia deferentially altered GR alternative exon1 mRNA variants in adult male offspring. Compared with the normoxia, the expression of exon 1_4_, 1_5_, 1_6_, 1_7_, and 1_10_ was significantly decreased while other exon1_S_ were unchanged in the heart of male offspring with prenatal hypoxia, which was similar to the expression of exon1_s_ in the fetal heart (Xiong et al., 2016). As for which exon1 form(s) make the major contribution remains elusive, and further study will be needed to investigate the role of the alternative GR promoters in this process.

Epigenetic gene regulation, especially the cluster (islands) of methylated cytosine, guanine, dinucleotide (CpG), established in embryogenesis and shortly after birth, plays an important role in the stability of specific gene silencing (Deaton and Bird, 2011). Many gene promoter regions containing several CpG dinucleotide are usually affected by changes of methylation. The functional consequences of promoter methylation on transcriptional regulation have been extensively documented including GR (Deaton and Bird, 2011; Xiong et al., 2016). The proximal CpG islands of GR gene, which located about 5 Kb upstream of the GR exon 2, was found to be highly structured and conserved in human and rodents (McCormick et al., 2000). This CpG-rich region encodes multiple alternative first exons that show remarkable similarity between human and rats (Nunez and Vedeckis, 2002; Turner and Muller, 2005; Turner et al., 2006, 2014). The role of epigenetic mechanisms, especially DNA methylation, in the regulation of GR expression by intrauterine hypoxia, and the methylation at promoter 1_4_, 1_5_, 1_6_, 1_7_ in newborn heart has been suggested in our previous study (Xiong et al., 2016). In the present study, we further determined methylation levels of critical CpGs in GR promoter by using an anti-mC antibody that specifically recognizes the methylated cytosine, and the general methylation level of each promoter was determined. In addition to promoter 1_4_, 1_5_, 1_6_, 1_7_, we found that the methylation levels of promoter 1_10_ were increased in the heart of adult male offspring. This data demonstrated that the epigenetic regulation of GR was shown to be highly continuity from newborn to adulthood after prenatal hypoxia. Since the distance between adjacent alternative promoters are very short, MeDIP may just reflect a general methylation level changes of alternative promoters of GR exon 1, but may not specify alternative promoters. However, the result of transcription factor binding assay by ChIP showed that maternal hypoxia significantly decreased transcription factor binding at the promoters of exon 1_4_, 1_5_, 1_6_, 1_7_, and 1_10_, providing support for the increased methylation level at these specific locations.

It has been documented that multiple transcription factor binding sites with CpG-rich regions locate at the GR promoter, and alternations of DNA methylation level determine GR expression (Suehiro et al., 2004; Turner et al., 2014; Xiong et al., 2016). We have determined transcription factor binding sites of GR promoter exon 1_s_, including CREs binding sites at promoter 1_4,_ 1_5_, and Sp1 binding sites at promoter 1_6_, 1_7_, 1_10_. Hypoxia induced CpG hypermethylation at those sites contributed to the decreased binding of transcription factors to the structure of GR gene in fetal heart (Xiong et al., 2016). In the present study, we found that prenatal hypoxia increased the methylation of promoter exon1_4-7_, 1_10_ in adult male offspring, disturbing transcription factor Sp1 binding to exon1_4_, 1_5_, and CRE binding to exon1_6_, 1_7_, 1_10_, resulting in reduced GR gene expression.

In summary, in the present study we revealed that prenatal hypoxia resulted in sex-differential GR gene suppression by promoter hypermethylation in the heart of adult offspring. Our previous study has determined that prenatal hypoxia induced GR repression in the developing heart. This study further establish the mechanistic link between prenatal hypoxia and epigenetic regulation of GR expression in adult offspring in rats. Thus, our finding highlighted the critical role of GR not only in developing heart after birth, but also in the hearts of adults.

## Author Contributions

JL, QM, ZX, and LZ contributed to the study design, drafting and preparation of the manuscript. JL and CD contributed to experiments and data collection and analysis. All authors read and approved the final manuscript.

## Conflict of Interest Statement

The authors declare that the research was conducted in the absence of any commercial or financial relationships that could be construed as a potential conflict of interest.
